# Genomic comparison between two *Inonotus hispidus* strains isolated from growing in different tree species

**DOI:** 10.3389/fgene.2023.1221491

**Published:** 2023-07-13

**Authors:** Qingchun Wang, Haiying Bao, Zhijun Li

**Affiliations:** ^1^ Key Laboratory for Development and Utilization of Fungi Traditional Chinese Medicine Resources, Jilin Agricultural University, Changchun, Jilin, China; ^2^ Key Laboratory of Edible Fungal Resources and Utilization (North), Ministry of Agriculture and Rural Affairs, Jilin Agricultural University, Changchun, Jilin, China

**Keywords:** *Inonotus hispidus*, whole genome sequencing, comparative genomic, CAZymes, functional annotation

## Abstract

*Inonotus hispidus* mainly growing in broad-leaved trees, including *Morus alba*, *Fraxinus mandshurica*, and *Ulmus macrocarpa* etc. The fruiting body of *I. hispidus* growing in *M. alba* (hereafter as MA) is used as a traditional Chinese medicine “*Sanghuang*”. However, differences between the genetic material basis of *I. hispidus* growing in other tree species have not been reported. Therefore, in this paper, the genomic comparison between MA and *I. hispidus* growing in *F. mandshurica* (hereafter as FM) were studied. The whole genome of MA monokaryon was sequenced by Illumina combined with Pac Bio platform. Next, genome assembly, genome component prediction and genome functional annotation were performed. Comparative genomics analysis was performed between FM monokaryon and MA monokaryon, using MA as the reference. The results showed that, MA had 24 contigs with a N50 length of 2.6 Mb. Specifically, 5,342, 6,564, 1,595, 383 and 123 genes were annotated from GO, KEGG, KOG, CAZymes and CYP450, respectively. Moreover, comparative genomics showed that, the coding genes and total number of genes annotated in different databases of FM were higher than that of MA. This study provides a foundation for the medicinal application of FM as MA from the perspective of genetic composition.

## 1 Introduction

Edible and medicinal fungus *Inonotus hispidus* (Bull.: Fr.) P. Karst belongs to Basidiomycota, Agaricomycetes, Hymenochaetales, Hymenochaetaceae, and *Inonotus*. It is distributed in China, Korea, Japan, Germany, Russia, Canada and other countries. Recent international medical studies reported that its pharmacological activities include anti-tumor, anti-oxidation, anti-inflammatory, liver protection, bacteriostasis, anti-virus, hypolipidemic and immune enhancement (Awadh et al., 2003; Benarous et al., 2015; Gründemann et al., 2016; Angelini et al., 2019; Li et al., 2019; Liu et al., 2019; Yang et al., 2019; Yang et al., 2020; Li and Bao, 2022a; Zhang et al., 2022a; Yang et al., 2022; Machado-Carvalho et al., 2023; Saka et al., 2023; Tang et al., 2023). *I. hispidus* has an excellent development space as medical and functional food in future. Its main hosts were *M. alba*, *F. mandshurica*, *U. macrocarpa*, *Z. jujuba* and *M. pumila* (Dai et al., 2000). FM is mainly distributed in Northeast China (Dai et al., 2000), whereas MA is prevalent in Northeast, Northwest, North, East and Southwest of China (Bao et al., 2020). According to the morphological characteristics, habitat and herbal research of MA, some Chinese scholars believe that the MA was positive source of traditional Chinese medicine “*Sanghuang*” (Bao et al., 2020). Historically, MA has been used as “*Sanghuang*” in both “The Ancient Course of the Yellow River” and southern Xinjiang regions. The types and enrichment degrees of chemical metabolites in the fruiting bodies of *I. hispidus* growing in five different tree species were reported to be significantly different using non-targeted metabolomics (Li and Bao, 2022b).

Previous research on *I. hispidus* has primarily focused on exploring the chemical composition, pharmacological action and domesticated culture, with little emphasis on whole genome sequencing. The rapid development of sequencing technology has greatly helped to solve the complex problems in the fungi biology. As of June 2023, 2,426 fungal genome projects had been completed and published on the JGI website (https://mycocosm.jgi.doe.gov/fungi/fungi.info.html), as well as *S. sanghuang* (Jiang et al., 2021), *I. hispidus* (Zhang et al., 2022b; Tang et al., 2022) and *G. lucidum* (Liu et al., 2012). These findings reveal novel insights for understanding gene network control, metabolite synthesis pathways, edible and medicinal fungal cultivation and breeding, contributing to large-scale fungi industrialization development bottleneck. As a result, there is no comprehensive explanation for the whole genome sequence and functional gene coding of *I. hispidus* growing in diverse tree species. This makes it difficult to elucidate the internal mechanism of different hosts at the molecular and genetic levels.

In order to establish a high-quality reference genome of MA and to facilitate subsequent analyses of its genome function and genetic material, as well as to determine whether FM has the value of development and utilization like MA. Therefore, in this study, the whole genome sequencing of MA would be carried out based on the SMRT (https://www.pacb.com/support/sofware-downloads/) (Ardui et al., 2018) and Illumina sequencing. The resulting genomic data of MA would be compared with the sequencing assembly, genome prediction, functional annotation and comparative genome analysis of FM. Furthermore, the similarities and differences in genetic material and metabolites of MA and FM would be explored, which would provide an important theoretical basis to research for transcriptomics, proteomics, metabolomics and development of *I. hispidus* growing in various tree species.

## 2 Materials and methods

### 2.1 Materials

**FIGURE 1:**
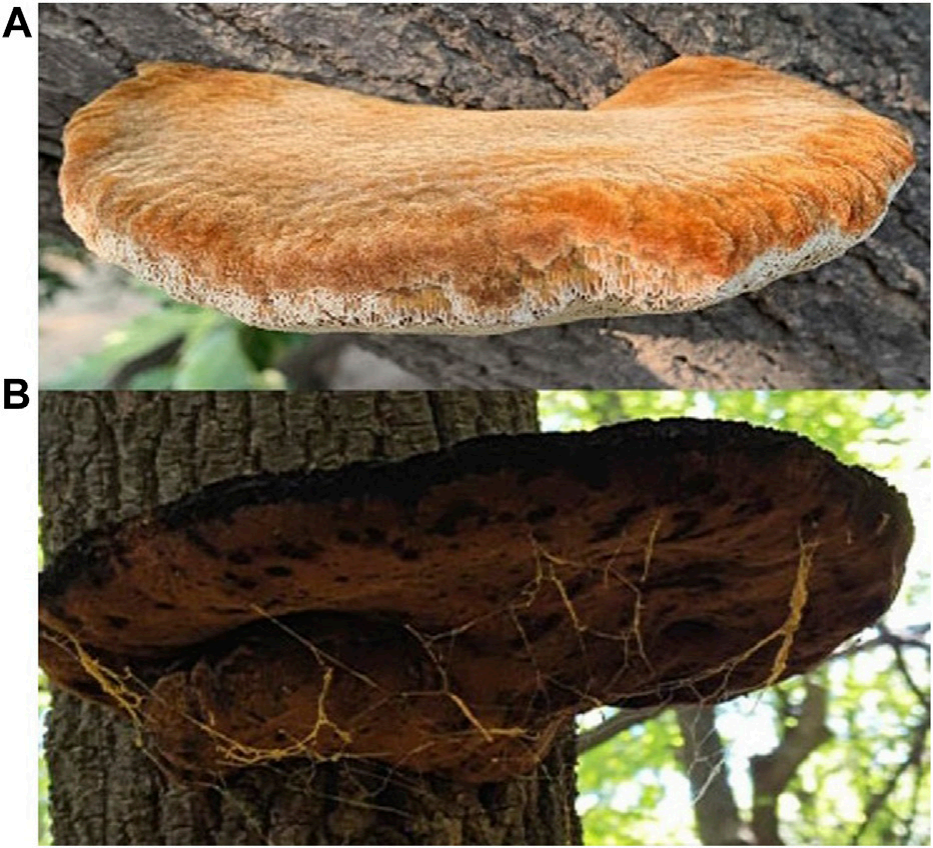
Fruiting bodies of *I. hispidus.* growing in two different trees. **(A)** Fruiting bodies of MA. **(B)** Fruiting bodies of FM.

In this study, *I. hispidus* growing in two different trees were collected from the field. MA (Figure 1A) was collected from Xiajin Ancient Mulberry Garden, Xiajin County, Shandong Province, China, 10 August 2020 (36°59′N, 115°11′E). FM (Figure 1B) was collected from Jingyuetan National Forest Park, Changchun City, Jilin Province, China, on 11 August 2020 (43°79′N, 125°46′E). The voucher specimen is deposited in the Herbarium of Mycology of Jilin Agricultural University (HMJAU), MA under No.58767 and FM under No.58769. We get the monokaryons of MA and FM by single spore isolation and used for whole genome sequencing. Then, we cultured the MA and FM monokaryon in PDA medium (Potato 200 g, Dextrose 20 g, Agar 20 g) in the dark at 28°C for 14 days. We verified isolation of MA and FM monokaryon were using an Axio Imager A2 fluorescence microscope (Zeiss). Subsequently, all samples were stored at −80°C until they were ready for genome-wide analysis.

### 2.2 Genome sequencing

#### 2.2.1 Extraction of genome DNA

Genomic DNA of MA and FM were extracted with the GP1 extraction method (Zhong et al., 2016). Added 25 mL GP1 lysate to the monokaryon of MA and FM, and immediately added 500 µL β-mercaptoethanol, mixed upside down, and lysed in a 65°C water bath for 30 min. Centrifuging at 12,000 rpm for 10 min, drawing the supernatant into a new 50 mL centrifuge tube; the equal volume of phenol, chloroform, isoamyl alcohol (25:24:1) was added to each tube, and the mixture was shaken and mixed for 5 min. Then centrifugation at 10°C, 12,500 rpm for 8 min, transfer the uppermost aqueous phase to a new 50 mL centrifuge tube. And added a double volume of chloroform, isoamyl alcohol and the mixture was inverted or shaken for 5 min; and the supernatant was transferred to a new 50 mL centrifuge tube after centrifugation at 10°C and 12,500 rpm for 8 min. The total volume of 3/4 volume of precooled isopropanol was added, mixed upside down for 10 times, incubated in dry ice at −80°C for at least 20 min, then centrifuged at 10°C, 12,000 rpm for 10 min, and the supernatant was discarded; each tube was added with 5 mL of new 75% alcohol to wash the precipitate, and centrifuged at 10°C, 12,000 rpm for 5 min to discard the supernatant carefully. Then 5 mL of 75% ethanol was added to wash the precipitate, and centrifuged at 10°C and 12,000 rpm for 3 min to discard the supernatant carefully. And centrifuge at 12,000 rpm for 2 min, absorb the liquid in the tube, and dry the fume hood for 3–5 min; then 200–400 µL of EB was added to dissolve the DNA, and 2 µL of RNase A was digested at 37°C for 25 min. Finally, the DNA sample of MA and FM were purified with the PowerClean Pro DNA, respectively. To ensure that the harvested DNA was detected by agarose gel electrophoresis and quantified by Qubit^®^ 2.0 fluorescence meter (Thermo Scientific).

#### 2.2.2 Library construction

Libraries for SMRT (Ardui et al., 2018) sequencing was constructed with an insert size of 20 kb using the SMRT bell TM Template kit (version 1.0.). Briefly, the process was that fragment and concentrate DNA, repair DNA damage and ends, prepare blunt ligation reaction, purify SMRT bell Templates with 0.45X AMPure PB Beads, size-selection using the BluePippin System, repair DNA damage after size-selection. Finally, the library quality was assessed using the Qubit^®^ 2.0 Fluorometer (Thermo Scientific) and the insert fragment size was determined using the Agilent 2100 (Agilent Technologies).

A total amount of 1 µg DNA per sample was used as input material for the DNA sample preparations. Sequencing libraries were generated using NEBNext^®^ Ultra™ DNA Library Prep Kit for Illumina (NEB, United States) following manufacturer’s protocol and index codes were added to attribute sequences to each sample. Briefly, the DNA sample was fragmented by sonication to a size of 350 bp, then DNA fragments were end-polished, a-tailed, and ligated with the full-length adaptor for Illumina sequencing with further PCR amplification. Finally, PCR products were purified (AMPure XP system) and libraries were analyzed for size distribution by Agilent2100 Bioanalyzer and quantified using real-time PCR.

#### 2.2.3 Sequencing

The whole genome of MA was sequenced using Illumina Nova Seq PE150 and Pac Bio Sequel, and FM using Illumina Nova Seq PE150. All of the above were sequenced at the Beijing Novogene Bioinformatics Technology Co., Ltd., China.

### 2.3 Genome assembly

The genome of MA and FM were obtained by assembling the clean data from the second-generation Illumina Nova Seq PE150 system by SOAP *de novo* software (Li et al., 2008). The gap close software was used to fill the gap in preliminary assembly results and removed the same lane pollution by filtering the reads with low sequencing depth (less than 0.35 of the average depth) to obtain the final assembly result of MA and FM. Then, fragments below 500 bp were filtered out and the final result was counted for genome prediction of MA and FM.

All of the data obtained from the Illumina Nova Seq PE150 system will pave the way for the Pac Bio Sequel genomic information of MA. In order to ensure the accuracy of the subsequent analysis results of MA, after filtering the low-quality reads (less than 500 bp) to obtain clean data, which from the Pac Bio Sequel platform were preliminary assembled using SMRT Link version 5.0.1 (Ardui et al., 2018). The long reads of MA were selected (more than 6,000 bp) as the seed sequence, and the other shorter reads were aligned to the seed sequence by Blasr. Then, by the variant Caller module of the SMRT Link software, the arrow algorithm was used to correct and count the variant sites in the initial genome sequence of MA. Finally, the integrity of sequence assembly of MA was evaluated by BUSCO (Benchmarking Universal Single Copy Orthologs, version 2.0) (Simão et al., 2015).

### 2.4 Genome component prediction and function annotation

Following the assembly process, bioinformatics software was employed to analyzed the genome components of MA and FM. Genome prediction predicts the number of coding genes, repeat sequences and non-coding RNAs. The obtained MA and FM genome-wide data were subjected to Augustus 2.7 program (Keller et al., 2011) *ab initio* prediction. The interspersed repetitive sequences, tandem repeat sequences, transfer RNA (tRNA) and rRNA were predicted by Repeat Masker (Saha et al., 2008) (http://www.repeatmasker.org/), TRF (Maddi et al., 2022) (Tandem repeats finder), tRNAscan-SE (Chan et al., 2021) and rRNAmmer (Lagesen et al., 2007) software, respectively. The sRNA, snRNA and miRNA were predicted by BLAST against the Rfam (Kalvari et al., 2018) database.

Genome function annotation results of MA and FM were compared using functional databases, including Gene Ontology (GO) (http://www.ebi.ac.uk/GOA, accessed on 7 August 2021) (Ashburner et al., 2000), Kyoto Encyclopedia of Genes and Genomes (KEGG) (https://www.kegg.jp/, accessed on 7 August 2021) (Kanehisa et al., 2006), Clusters of Orthologous Groups (KOG) (http://www.ncbi.nlm.nih.gov/COG, accessed on 7 August 2021) (Galperin et al., 2015), Non-Redundant Protein Database (NR) (ftp://ftp.ncbi.nlm.nih.gov/blast/db/, accessed on 7 August 2021) (Li et al., 2002), Transporter Classification Database (TCDB) (http://www.tcdb.org, accessed on7 August 2021) (Saier et al., 2021), Cytochromes P450 (CYP450) (http://drnelson.utmem.edu/CytochromeP450.html, accessed on 7 August 2021) (Kohler et al., 2015), Swiss-Prot (http://www.expasy.org/sprot/and http://www.ebi.ac.uk/swissprot/, accessed on 7 August 2021) (Bairoch and Apweiler, 2000) and Protein Families Database of Alignments and Hidden Markov Models (Pfam) (http://pfam.xfam.org/, accessed on 7 August 2021) (Eddy, 1998). A whole genome Blast (Reiner et al., 2018) search (E-value less than 1e^−5^, minimal alignment length percentage larger than 40%) was performed against above eight databases. The predict secretory protein, gene cluster of secondary metabolites and carbohydrate-active enzyme were predicted using the Carbohydrate-active Enzyme Database (CAZymes) (http://www.cazy.org/, accessed on 7 August 2021) (Cantarel et al., 2008), Signal P database (Petersen et al., 2011) and antiSMASH (Blin et al., 2021) database.

### 2.5 Comparative genomics analysis

Genomic alignment between the sample of FM genome and reference genome of MA were performed by MUMmer (Marçais et al., 2018) and LASTZ (Aljouie and Zhong, 2020) tools. Genomic synteny was analyzed based on the alignment results. InDels, SNPs and SVs were found by the genomic alignment results among samples by the MUMmer and LASTZ. Comparative analysis of genome sequence, component predictions and genome function annotations were the same as Sections 2.2–2.4.

## 3 Results

### 3.1 Genome sequence and component prediction

**FIGURE 2:**
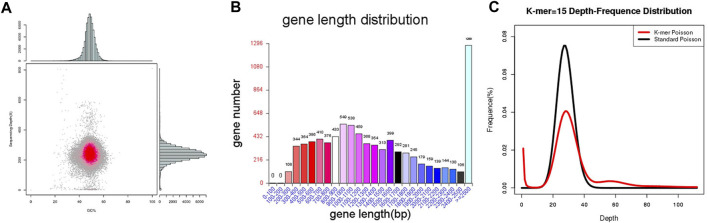
Genome sequencing, assembly and prediction of MA. **(A)** Statistical chart of correlation analysis between MA GC content and sequencing depth. The horizontal axis represents the GC content, the vertical axis represents the sequencing depth, the right is the sequencing depth distribution, and the upper is the GC content distribution. **(B)** The gene length distribution of MA. The horizontal axis shows the gene length, and the vertical axis shows the corresponding gene number. **(C)** K-mer analysis diagram of MA.

Through the Pac Bio Sequel platform, it was found that MA generated 817,989 Mb Pac Bio-data. The 34.14 Mb genome sequence was assembled from 24 contigs with N50 length was 2.6 Mb. Of the 24 contigs, the longest one was 4.5 Mb in length. The analysis of GC content and read coverage depth of the MA assembly sequence revealed no significant contamination of the sample DNA. However, scattered points were observed in the region with a GC content ranging from 20% to 40%. These anomalies could potentially be attributed to the presence of mitochondrial DNA in the fungal genomic samples (Figure 2A). The sequence and feature summary of MA *de novo* assembly results demonstrated that high-quality sequence have been obtained (Supplementary Table S1). Using BUSCO software, we identified 94.8% (277/290) of well-known fungal complete and single-copy in MA assembly, which further illustrated a high accuracy and integrity of MA monokaryon (Supplementary Table S2). This Whole Genome Shotgun project has been deposited in GenBank under the accession number JASXRQ000000000. The final genome assembly results and related data of MA were submitted to the NCBI Bio Project PRJNA973857, Bio Sample SAMN35152529 and SRA accession number was SUB13375186, respectively.

**FIGURE 3:**
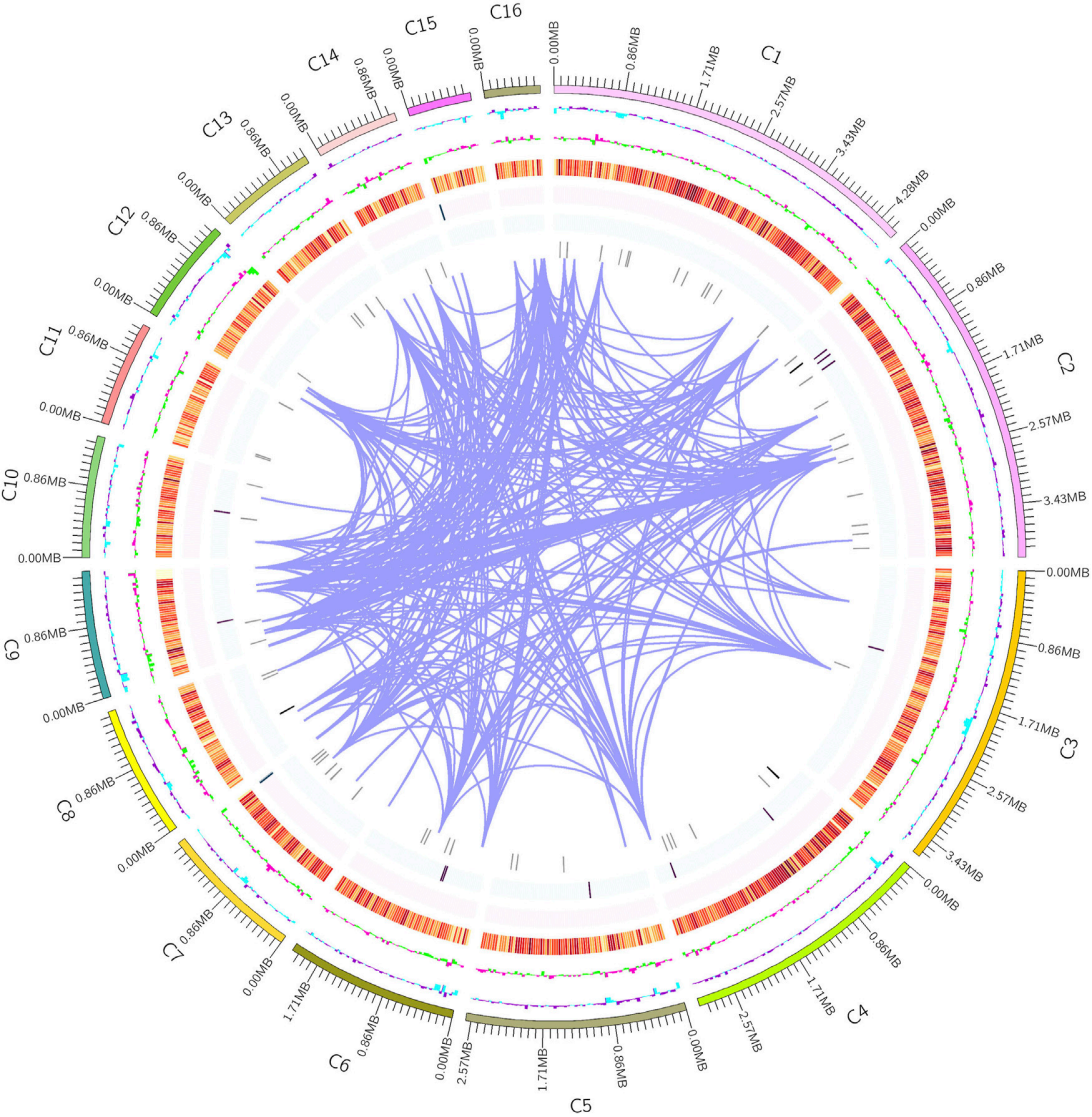
Circular whole genome diagram of MA. The outermost layer is the position coordinates. From the outer circle to the inner circle is GC content (purple: > mean value, blue: < mean value). GC skew (the specific algorithm= (G−C)/(G + C); pink: > 1, green: < 1). Gene density (four circles were taken inward from orange, representing the numerical value of coding genes, rRNA, snRNA, and tRNA, respectively). Genome chromosome duplication (regions with similarity greater than 90% of 8 kb sequences were connected by purple lines).

MA had a total number of 8,356 protein-coding genes were predicted with a total gene length of 12,839,852 bp, accounting for 37.61% of the whole genome size (34,136,716 bp); and an average gene length of coding genes was 1,537 bp, most of which ranged from 300 bp to 1,700 bp (Table 1; Figure 2B). In the prediction of repeat sequences, the interspersed and repeats were 2,761 and 5,626, respectively. For interspersed, the LTRs, DNAs, LINEs, SINEs and rolling circles contained 1,901, 493, 3,146 and 33, respectively. Moreover, 14 unknowns with a total length of 1,371 bp are to be further explored. For repeats, the TRs, minisatellite DNAs and microsatellite DNAs contained 3,045, 1,985 and 596, respectively (Supplementary Table S3). Regarding RNAs, 88 tRNAs, 9 rRNAs and 13 snRNAs were predicted (Supplementary Table S4). Based on the aforementioned information, the detailed circular genome diagram information of MA was visually represented using Circos (Krzywinski et al., 2009) software (Figure 3).

**TABLE 1:** Statistics of gene prediction results of MA.

Parameter	Value
Genome size	34,136,716 bp
Gene number	8,356
Gene total length	12,839,852 bp
GC content (%)	51.62
Gene length/Genome (%)	37.61
Gene average length	1,537 bp
Gene internal length	21,296,864 bp
Gene internal GC content	46.59

### 3.2 Genome function annotation

#### 3.2.1 Gene general annotation

**FIGURE 4:**
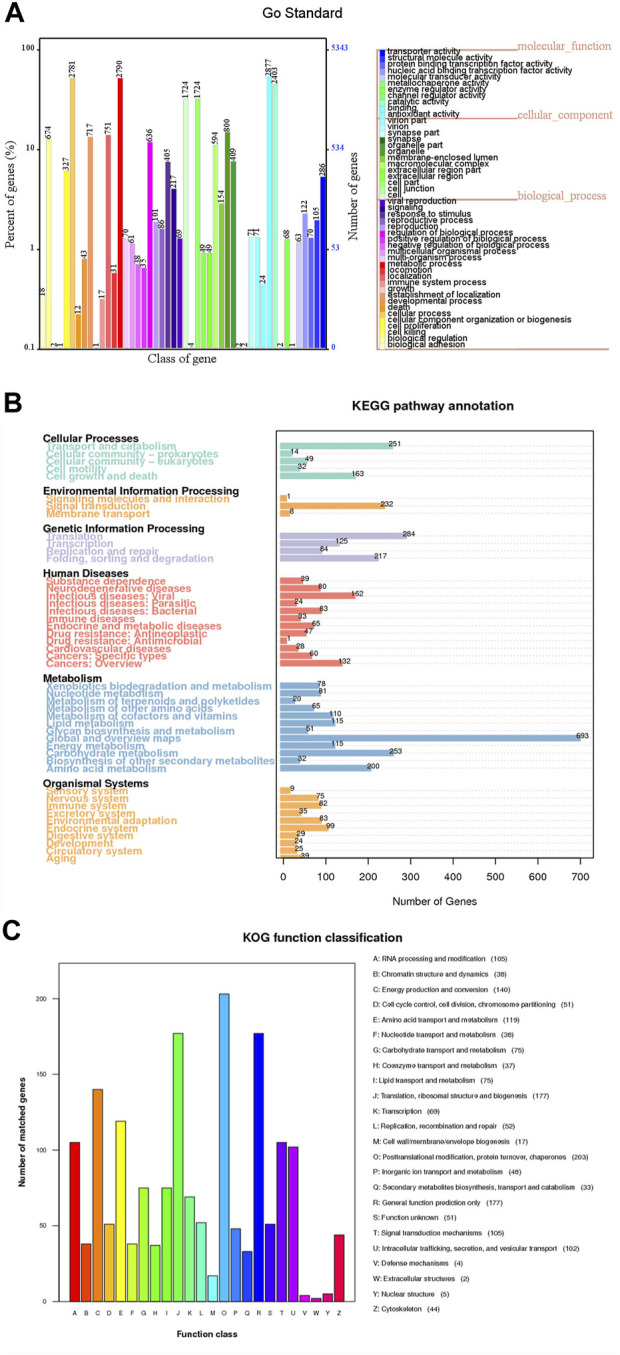
Different public database gene annotation map of MA. **(A)** GO annotation. The horizontal axis represents the GO functional classification on the sample annotation, the right vertical axis represents the number of genes on the annotation, and the left vertical axis represents the percentage of the number of genes on the annotation to all coding genes. **(B)** KEGG (Level 1) annotation. The number on the bar graph represents the number of genes on the annotation. **(C)** KOG annotation. The horizontal axis represents the KOG functional type, and the vertical axis represents the number of genes on the annotation.

Different public databases were used to predict the number of genes enriched in each functional category (Supplementary Table S5). According to the GO database, MA obtained 5,342 genes accounted for 17.60% of the genome and were classified into 3 categories and 48 subcategories. There were mainly distributed in six functional subcategories, binding (2,877), metabolic process (2,790), cellular process (2,781), catalytic activity (2,403), cell (1,724) and cell part (1,724) (Figure 4A).

MA had 6,564 genes were annotated in the KEGG database, among them 1,864 genes were enriched in 366 KEGG pathways (Figure 4B). The most and least enriched functional annotations were metabolism (1,813) and environmental information processing (241), respectively; with the top three coding genes being global and overview maps (693), translation (284) and carbohydrate metabolism (253) (Supplementary Table S6). There were 107 metabolism pathways, with purine metabolism (59), oxidative phosphorylation (56), pyrimidine metabolism (51), amino sugar and nucleotide sugar metabolism (46), starch and sucrose metabolism (43), pyruvate metabolism (37), glycolysis/gluconeogenesis (36), cysteine and methionine metabolism (34) and arginine and proline metabolism (33) and so on. Furthermore, the main enriched signaling pathways were MAPK signaling pathway-yeast (54), mTOR signaling pathway (43), PI3K-Akt signaling pathway (29), Ras signaling pathway (27), MAPK signaling pathway (25), FoxO signaling pathway (24) and other effective pathways (Supplementary Table S7).

The data with the KOG protein database to obtain the phylogenetic relationship classification of fungal complete genome-encoded proteins (Figure 4C). There were 1,595 genes annotated in the KOG database. Posttranslational modification, protein conversion, and chaperones (203) coding genes were the most abundant. The abundant number of classes in the KOG groups were translation, ribosomal structure and biogenesis (177), general function prediction only (177), energy production and conversion (140) and amino acid transport and metabolism (119).

#### 3.2.2 Gene special annotations

We used multiple databases, including NR, Pfam, Swiss-prot, TCDB, CAZymes, secretory proteins, CYPs and gene clusters of secondary metabolites to further elucidated the genomic functions of MA. In MA, 7,177 (23.67%) genes were assigned to the NR database. The top three species were *S. baumii*, *F. mediterranea* and *P. pouzarii* (Supplementary Table S8). A total of 5,342 genes of MA were annotated to the Pfam database. Moreover, 6.89% (2,090) of the predicted genes were annotated to the Swiss-prot database. There were 365 (4.37%) genes annotated in TCDB database. Among TCDB, the order of proportion from large to small were the primary active transporters (122, 33.42%), electrochemical potential-driven transporters (119, 32.60%), incompletely characterized transport systems (45, 12.32%), channels/pores (42, 11.50%), accessory factors involved in transport (29, 7.95%), group transporters (8, 2.19%) and transmembrane electron carriers were not included.

The genome of macrofungus contains CAZymes that play an essential role in facilitating the acquisition of complex carbohydrate metabolism. There were 418 genes annotated in CAZymes database (Table 2). Most of these genes encode glycoside hydrolases (GHs) (185, 44.26%), glycosyltransferases (GTs) (79, 18.90%), auxiliary activity (AAs) (63, 15.07%), carbohydrate-binding modules (CBMs) (47, 11.24%), carbohydrate esterases (CEs) (33, 7.89%) and polysaccharide lyases (PLs) (11, 2.63%). From the CAZymes database, a total of 148 CAZymes families were detected in MA monokaryon (Supplementary Table S9). In the MA genome, GHs were distributed in 65 families; among the main enzymes of lignocellulose degradation, cellulase identified 12 families with GH1-GH3, GH-7, GH9, GH10, GH12, GH51, GH74, AA9 and AA16. Hemicellulose was mainly distributed in 15 families with GH1-3, GH6-7, GH9, GH12, GH17, GH27, GH29-31, GH35, GH51 and GH74. Pectinase was mainly distributed in GH2-3, GH9, GH12, GH28, GH53, GH74, GH78, PL14, CE8 and other 10 families. Ligninase was mainly distributed in AA1-2 2 families. CBMs include CBM1, CBM12, CBM13, CBM20, CBM21 and CBM50 families. CEs were divided into CE1, CE4, CE8-9, CE12 and CE15-16 8 families. PLs were mainly distributed in 8 families with PL14, PL35 and PL38 (Supplementary Table S10).

**TABLE 2:** Comparative analysis of genomic components and comparison of different databases for MA and FM.

Characteristic	Type	Gene number	
		MA	FM
Comparison results for genomic components	Genome size (bp)	34,136,716	31,430,498
	Gene number	8,356	9,167
	Gene total length (bp)	12,839,852	12,719,201
	Gene average length (bp)	1,537	1,387
	Gene length/Genome (%)	37.61%	40.47%
Comparison results for TCDB database	Channels/pores	42	38
	Electrochemical potential-driven transporters	119	118
	Primary active transporters	122	127
	Group translocators	8	8
	Transmembrane electron carriers	0	0
	Accessory factors involved in transport	29	33
	Incompletely characterized transport systems	45	50
Comparison results for CAZymes database	Glycoside hydrolases (GHs)	185	192
	Glycosyltransferases (GTs)	79	90
	Auxiliary activity (AAs)	63	64
	Carbohydrate-binding modules (CBMs)	47	50
	Carbohydrate esterases (CEs)	33	27
	Polysaccharide lyases (PLs)	11	12
Comparison results for secretory protein prediction	Signal protein	467	558
	TMHMM protein	1,291	1,551
	Secreted protein	333	384
Comparison results for cytochromes P450 database	P450, CYP52	4	5
	E-classP450, CYP2D	1	1
	Undeterminded	15	22
	CytochromeP450	9	14
	E-classP450, group I	84	82
	E-classP450, group IV	7	8
	Pisatindemethylase-like	3	3
Comparison results for gene clusters of secondary metabolites	Terpene	8	11
	NRPS-like, T1PKS	2	2
	NRPS	1	1
	NRPS-like	3	4
	T1PKS	1	1

Next, 467, 1,291 and 333 proteins with signal peptide structure, transmembrane structure and signal peptide structure without transmembrane structure were obtained of MA, respectively. A total of 123 genes from MA were assigned to encode CYPs. The highest (84, 68.29%) had E-class P450 group I annotations (Table 2). Moreover, 20 genes were involved in the “Metabolism of xenobiotics by cytochrome P450” (Supplementary Table S11) and 18 genes were involved in the KEGG sub-pathway “Drug metabolism-cytochrome P450” (Supplementary Table S12). A total of 15 gene clusters were obtained from MA, which were 8 terpenes, 1 NRPS, 1 T1PKS and 5 other clusters. Additionally, we detected the MA gene in the “Terpenoid backbone biosynthesis (map 00900)” pathway and found 12 key enzymes distributed in the MVA pathway (Table 3). Hexaprenyl-diphosphate synthase, prenylcysteine oxidase/farnesylcysteine lyase, farnesyl diphosphate synthase and protein farnesyltransferase/geranylgeranyltransferase type-1 subunit alpha was encoded by double-copy genes. In contrast, the other 8 enzymes were encoded by a single-copy gene (Supplementary Table S13).

**TABLE 3:** Core enzymes involved in terpenoid biosynthesis of MA.

Gene name and definition	EC No.	KO term	Gene ID
hexPS, COQ1; hexaprenyl-diphosphate synthase	2.5.1.82, 2.5.1.83	K05355	A1965
PCYOX1, FCLY; prenylcysteine oxidase/farnesylcysteine lyase	1.8.3.5, 1.8.3.6	K05906	A2517
DHDDS, RER2, SRT1; ditrans, polycis-polyprenyl diphosphate synthase	2.5.1.87	K11778	A3541
HMGCR; hydroxymethylglutaryl-CoA reductase (NADPH)	1.1.1.34	K00021	A3675
FDPS; farnesyl diphosphate synthase	2.5.1.1, 2.5.1.10	K00787	A4491
E2.3.3.10; hydroxymethylglutaryl-CoA synthase	2.3.3.10	K01641	A4708
E2.3.1.9, atoB; acetyl-CoA C-acetyltransferase	2.3.1.9	K00626	A5693
STE24; STE24 endopeptidase	3.4.24.84	K06013	A7679
FNTB; protein farnesyltransferase subunit beta	2.5.1.58	K05954	A7968
MVD, mvaD; diphosphomevalonate decarboxylase	4.1.1.33	K01597	A8252
FNTA; protein farnesyltransferase/geranylgeranyltransferase type-1 subunit alpha	2.5.1.58 2.5.1.59	K05955	A1633
E2.7.4.2, mvaK2; phosphomevalonate kinase	2.7.4.2	K00938	A1934

### 3.3 Comparative genomics analysis

#### 3.3.1 Comparative analysis of genome sequence and component prediction

Comparative genomic analysis of genome sequence, it was found that raw reads, clean reads and filtered reads of FM were 5,595, 4,507 Mb and 46.14%, respectively, above numbers were all higher than ones of MA. Then, the number and the depth of K-mer of FM were also higher than those of MA (Figure 2C), and the former number was 885,330,899 and the latter number was 22.92. But the number of revised size and repeat rate of FM were lower than ones of MA (Supplementary Table S1). By comparing the genome component analysis, it was demonstrated that the genome size, gene total length and gene average length of FM were lower than those of MA, which were 31,430,498, 12,719,201, and 1,387 bp, respectively. Genome component prediction comparison showed that protein-coding genes of FM were higher than MA, reaching 9,167 (Table 2). However, the repeat sequences results of FM were lower than those of MA (Supplementary Table S3), contained only 71 tRNAs (Supplementary Table S4).

#### 3.3.2 Comparative analysis of genome function annotation

The numerical comparison of MA and FM in the GO functional database showed that more genes were enriched in binding, metabolic process and cellular process, and they were annotated to three types of biological functions, indicating high similarity. Compared with MA, FM lacked three subcategories such as growth, synapse and synapse part. And a total number of 5,555 genes were obtained of FM, accounting 17.55% for all the genes, and it was found that cellular component and molecular function were higher than MA, while biological process was lower than MA (Supplementary Figure S1A). Compared with KEGG annotations of MA and FM, the results indicated that the total number of FM (6,943, 21.93%) genes were higher than that of MA and also categorized into 6 levels and 46 categories; and the number of FM genes associated with most genome functions was not significantly different from the number of genes for MA. In addition, its metabolism is also mainly related to global and overview maps (724), carbohydrate metabolism (256), amino acid metabolism (203), energy metabolism (125) and lipid metabolism (123) (Supplementary Figure S1B). However, due to the preliminary nature of the FM measurement, a more in-depth exploration is required to elucidate specific metabolic pathways and identify enriched genes, which can be further compared with MA. In view of MA and FM in the KOG functional database also have high similarity. The number of genes annotated by MA were less than that of FM genome (1,641), but there was no difference in KOG types (Supplementary Figure S1C).

Given that the TCDB database annotation, the total number of FM genes were higher than MA, reaching 374. There was no markedly difference in types (Table 2). Like MA, primary active transporters (127, 33.95%) were the most enriched (Table 2). FM contained more CAZymes genes, reaching 394. Among them, glycoside hydrolases (GHs) (192, 48.73%), glycosyltransferases (GTs) (90, 22.84%), auxiliary activity (AAs) (64, 16.24%), carbohydrate-binding modules (CBMs) (50, 12.69%) and polysaccharide lyases (PLs) (12, 3.05%) were higher than MA, but carbohydrate esterases (CEs) (33, 8.38%) was lower than MA (Table 2). In addition, the total number of FM secretory protein prediction were higher than that of MA (Table 2).

Cytochromes P450 database comparison showed that, E-class P450 group I (82, 60.74%) were high expression enrichment in FM genome, followed by Undeterminded (22, 16.30%) and Cytochrome P450 (14, 10.37%) (Table 2). There were 19 gene clusters of secondary metabolites annotated in FM, including 11 terpenes and 4 NRPS-likes. Both samples contained five kinds of gene clusters of secondary metabolites, further revealing high similarity; it also contains two NRPS-like, T1PKS genes, one NRPS gene and one T1PKS gene. The comparative analysis of the above genome functional annotations will provide a potential reference for whether FM is as medicinal as MA (Table 2).

#### 3.3.3 Collinearity, InDel, SNP, and SV analysis

**FIGURE 5:**
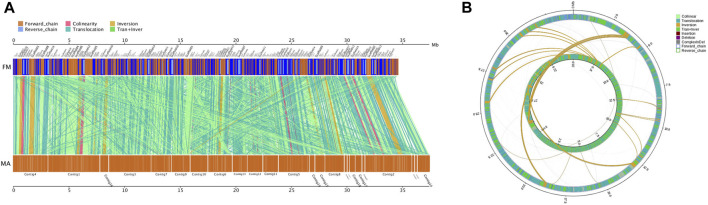
Comparative genomics analysis of MA and FM. **(A)** Parallel collinearity comparison between MA and FM. **(B)** Genome-wide structural variation type pairing map. The inner ring and the outer ring were FM and MA, respectively.

The results of collinearity analysis between MA and FM showed that, the total base length of the MA sequence was 29,119,278 bp, which accounted for 92.65% of the total gene length of 31,430,498 bp. There were 6,499 collinearity blocks in MA and FM. MA and FM had many similar translocations, translocation and inversion alignments, indicating that high collinearity relationship between them (Figure 5A; Supplementary Table S14). There were 8,356 InDels, including 275 gene coding regions, 124 insertions and 151 deletions identified between FM and MA. These InDels affected 11 CDS regions, 3 of which led to frameshifts, and 1 InDel resulted in a premature stop (Supplementary Tables S15, S16). There were 228,256 SNPs found between FM and MA, of which 66,280 SNPs occurred in the gene coding regions. The CDS SNPs and intergenic FM were 0, 57, 62, 63, 227, 41,037, 24,834, 66,280 and 161,976, respectively. These values represented 0%, 0.0250%, 0.0272%, 0.0276%, 0.0994%, 17.9785%, 10.8799%, 29.0376% and 70.9624% of the total SNPs (Supplementary Tables S17, S18). FM had 9,020 SVs, of which 3,799 translocations account for 42.1% of the total SVs; 179 inversions account for 1.9%; 3,462 translocations and inversions account for 38.3%; 336 insertions account for 3.7%; 1,143 deletions account for 12.6%; and 98 complex InDels contribute for 1% (Figure 5B; Supplementary Table S19).

## 4 Discussion

Fungi has been used as functional food and traditional medicine for a long history in China. Among them, “*Sanghuang*” is a well-known edible and medicinal fungus, which is used as health preservation and treatment of diseases. It was recorded in several ancient traditional Chinese herbal books, including *Shennong’s Classic of Materia Medica* and *Compendium of Materia Medica* (Bao et al., 2020). At present, the whole genome sequencing of *I. hispidus* growing in different tree species has not been reported. Hence, in this paper, two strains of *I. hispidus* growing in *M. alba* and *F. mandshurica* were chosen to reveal differences of genome sequence and epigenetics*.* The whole genome of MA monokaryon was sequenced by the combination of the Illumina and Pac Bio platform. Then using MA as a reference, comparative genomics between MA monokaryon and FM monokaryon was analyzed. The result demonstrated that, MA had 8,356 protein-coding genes, 24 contigs with a contig N50 size of 2.6 Mb and a genome-wide map of 34.14 Mb was drawn. The average genome size of most fungi is about 40 Mb (Duan et al., 2022), and the assembled genome size obtained by MA is basically in line with the expected results.

According to the results of genome annotation, MA obtained 30,321 genes from different public databases, but a large number of predicted genes could not match the public database. As we know, KEGG is the essential public database about pathways. On the basis of KEGG functional annotation, MA were primarily connected with carbohydrate metabolism and amino acid metabolism. In light of pathways analysis, the most significant biochemical metabolic pathways and signal transduction pathways of MA were relevant to metabolites. By further analysis of metabolic pathways, MA were found that principally involved in purine metabolism, oxidative phosphorylation, pyrimidine metabolism, amino sugar and nucleotide sugar metabolism, starch and sucrose metabolism, pyruvate metabolism and some other secondary metabolic pathways. There were 31 signal transduction pathways, including MAPK signaling pathway-yeast, mTOR signaling pathway, PI3K-Akt signaling pathway and so on. MA contains 14 xenobiotics biodegradation and metabolisms. It was testified that MA has a strongly primary metabolic process and powerful ability to degrade aromatic compounds. Furthermore, CAZymes as a matrix-degrading enzyme that plays an extremely critical role in maintaining the metabolism and circulation of natural substances. MA had a great deal of genes encoding cellulase, hemicellulase, glucan degradation related enzymes, chitinase (EC 3.2.1.14), α-amylase (EC 3.2.1.-) and peptidoglycan degradation related enzymes. It was announced that MA can not only degrade plant polysaccharides, but also degrade N-acetylamino polysaccharides, and can destroy the integrity of cell wall structure by cooperating with glucanase, cellulase, peptidoglycanase and chitinase to maintain the growth and senescence of pileus (Ding et al., 2007; Tao et al., 2013). Simultaneously, other cell wall structure-related enzyme genes also further denoted that MA has the potential to specifically degrade the host cell wall to achieve the effect of biocontrol.

In addition, CYPs belong to the heme-containing monooxygenase superfamily. The functional and evolutionary diversity of fungal CYPs makes the classification of fungal CYPs more complex, mainly associated with the biosynthesis and metabolism of fungal endogenous substances such as fatty acids, sterols, terpenes. In the field of pharmacology and therapeutics, the secondary metabolites of medicinal fungi have been reported to have anti-inflammatory and anti-viral effects (Alves et al., 2012; Alves et al., 2013), usually located in the adjacent gene clusters (Keller et al., 2005), and can be found through gene mining. Gene clusters of secondary metabolites with TS, NRPS and PKS. It was found that all known fungal siderophores were synthesized by NRPS (Haas et al., 2008). Like most basidiomycetes, MA contains a small amount of NRPS and T1pks genes, demonstrated that MA has the ability to resist the stress of the external environment and maintain its own growth and metabolism. Among fungi, one of the largest groups of corroborate bioactive natural products were terpenoids, which were composed of isoprene, mainly derived from the mevalonate pathway (Miziorko, 2011; Quin et al., 2014; Adamek et al., 2017), and had a variety of pharmacological activities. For example, the triterpenoid ganoderic acids has been reported to have anti-tumor and immunomodulatory effects (Shi et al., 2010). Twelve key enzymes involved in the MVA pathway were identified from the “Terpenoid backbone biosynthesis (map 00900)” pathway, revealing that the biosynthesis of MA terpenoids is mainly formed by the MVA pathway like most fungi. Among them, lanosterol synthase (LSS) can catalyze the cyclization of (3S)-2,3-0xidosqualene to produce lanosterol. Various enzymes act downstream to convert precursors into ergosterol and other sterols in the fungus. Notably, the catalytic derivatization of these compounds is heavily reliant on the involvement of CYPs enzymes, which play a pivotal role in modifying the lanosterol skeleton (Benveniste, 2004). In the pursuit of identifying potential triterpenoid biosynthesis genes in MA, a single copy of the LSS gene (ERG7; K01852; EC 5.4.99.7) was investigated. Consequently, it is postulated that diverse structural types of sterols and triterpenes may be produced through the action of different enzymes acting upon the LSS gene, as evidenced by the presence of various families of MA CYPs and eight triterpene-related gene clusters.

Moreover, comparative genomics as a research method to comprehend the function, expression mechanism and species evolution of genes by comparing the gene structure and distribution of different genome. During the evolutionary trajectory of fungi, genetic variations such as gene deletions and acquisitions can occur as a result of environmental selection, ultimately influencing the fungal adaptive capacity. In this study, we conducted a comparative analysis of the genome structure and function of FM, utilizing MA as the reference genome. It was worth noting that, the genome composition of FM exhibited distinctions from MA in terms of genome size, the number of coding genes, repeat sequences and non-coding RNAs. These differences suggest that FM may possess enhanced environmental adaptability, implying a potential expansion of its adaptive repertoire throughout its evolutionary history. Comparison of GO, KEGG, KOG and CAZymes results showed that, there was no apparently difference in the function of genes enriched with MA. In accordance with gene clusters, FM contains 11 terpenes. Although, FM and MA types of genome function annotation were high similarity, the specific intrinsic active substances of FM need further exploration. As two common InDels and SNPs markers for genetic polymorphism analysis, it can thorough understand the genetic polymorphism of different species (Santos et al., 2018). A significant number of InDels and SNPs sites were identified between MA and FM, which provided a basis for the subsequent genetic map construction and phylogenetic research of *I. hispidus*. Since accurate screening and functional verification of candidate genes for FM are currently lacking, it is crucial to prioritize comprehensive functional verification of candidate sites. This will enable us to gain clues into the regulatory mechanisms underlying these genes and enhance our understanding of FM genetic characteristics. Through the implementation of gene knock-out experiments, we can examine alterations in the intrinsic activity and ascertain potential significant modifications in the target peak of the active product. The primary objective of the research is to understand the genetic composition of MA and to determine whether FM has the value of development and utilization like MA by comparative genomics analysis. In the future, we would continue to collect *I. hispidus* growing in other tree species and analyze the influence of different tree species on these strains by comparative genomics and transcriptomics.

## 5 Conclusion

In this study, a comprehensive overview of MA was conducted at the genetic level, and a comparative genomics analysis was performed for FM to compare differences in genetic materials and whether it has potential medicinal value as MA. A 34.14 Mb genome-wide map of MA was drawn by sequencing and assembly. Additionally, 9 major secondary metabolic pathways and 31 signal transduction pathways were obtained in MA. Although, comparative genomics analysis showed that FM had high similarity with MA, the total number of genes annotated in different databases of FM were higher than MA, including 11 terpenes, which provided new insights into the correlation of FM phenotypic characteristics and genetic mechanisms. It also provided a foundation for the development and utilization of *I. hispidus* growing in different tree species from the aspect of genomics research.

## Data Availability

The datasets presented in this study can be found in online repositories. The names of the repository/repositories and accession number(s) can be found below: NCBI, BioProject ID: PRJNA973857.
