# Supplementary material for: Genomic comparison between two Inonotus hispidus strains isolated from growing in different tree species
Source: Front Genet. 2023 Jul 13;14:1221491. doi: 10.3389/fgene.2023.1221491 (PMC10372432; doi:10.3389/fgene.2023.1221491)
Supplement: Supplementary file 1 [file DataSheet1.PDF]

Supplementary Material

Comparative Genomics Study of *Inonotus hispidus* Parasitize on Two Tree Species Based on The Whole Genome

Qingchun Wang[1,2], Haiying Bao[1,2*], Zhijun Li[1,2]

*** Correspondence:** Haiying Bao: baohaiying@jlau.edu.cn

1 Supplementary Figures and Tables

1.1 Supplementary Figures

Supplementary Figure 1. Different public database gene annotation map of FM. (A) GO annotation. The horizontal axis represents the GO functional classification on the sample annotation, the right vertical axis represents the number of genes on the annotation, and the left vertical axis represents the percentage of the number of genes on the annotation to all coding genes. (B) KEGG (Level 1) annotation. The number on the bar graph represents the number of genes on the annotation. (C) KOG annotation. The horizontal axis represents the KOG functional type, and the vertical axis represents the number of genes on the annotation.

1.2 Supplementary Tables

Supplementary Table 1. *De novo* assembly and characteristic information of MA and FM.

Supplementary Table 2. BUSCO assembly and characteristic information of MA.

Supplementary Table 3. Comparison results for repeats sequences of MA and FM.

Supplementary Table 4. Comparison results for ncRNA of MA and FM.

Supplementary Table 5. Comparison of gene function annotations in different databases of MA and FM.

Supplementary Table 6. KEGG gene function annotations of MA.

Supplementary Table 7. KEGG catalog maps of MA.

Supplementary Table 8. Comparison of the top 20 species annotations between MA and FM NR databases.

Supplementary Table 9. CAZymes function annotations of MA.

Supplementary Table 10. Genetic information of all CAZYmes family classifications of MA.

Supplementary Table 11. Statistical results of 20 genes annotated Metabolism of xenobiotics by cytochrome P450 [PATH: ko00980].

Supplementary Table 12. Statistical results of 18 genes annotated Drug metabolism - cytochrome P450 [PATH: ko00982].

Supplementary Table 13. Core enzymes involved in terpenoid biosynthesis.

Supplementary Table 14. Collinearity comparison coverage statistics of MA and FM.

Supplementary Table 15. FM InDel cds.

Supplementary Table 16. FM InDel cds information.

Supplementary Table 17. FM SNP cds.

Supplementary Table 18. FM SNP cds information.

Supplementary Table 19. SV analysis of MA and FM.
